# Atypical patterns of tone production in tone-language-speaking children with autism

**DOI:** 10.3389/fpsyg.2022.1023205

**Published:** 2022-11-03

**Authors:** Kunyu Xu, Jinting Yan, Chenlu Ma, Xuhui Chang, Yu-Fu Chien

**Affiliations:** ^1^Institute of Modern Languages and Linguistics, Fudan University, Shanghai, China; ^2^School of Foreign Languages, Hunan University, Changsha, China; ^3^Cangzhou Research Centre for Child Language Rehabilitation, Cangzhou Normal University, Hebei, China; ^4^Department of Chinese Language and Literature, Fudan University, Shanghai, China; ^5^College of Foreign Languages and Literature, Fudan University, Shanghai, China

**Keywords:** autism spectrum disorder, Mandarin Chinese, tone production, F0, atypical tone

## Abstract

Speakers with autism spectrum disorder (ASD) are found to exhibit atypical pitch patterns in speech production. However, little is known about the production of lexical tones (T1, T2, T3, T4) as well as neutral tones (T1N, T2N, T3N, T4N) by tone-language speakers with ASD. Thus, this study investigated the height and shape of tones produced by Mandarin-speaking children with ASD and their age-matched typically developing (TD) peers. A pronunciation experiment was conducted in which the participants were asked to produce reduplicated nouns. The findings from the acoustic analyses showed that although ASD children generally produced both lexical tones and neutral tones with distinct tonal contours, there were significant differences between the ASD and TD groups for tone height and shape for T1/T1N, T3/T3N, and T4/T4N. However, we did not find any difference in T2/T2N. These data implied that the atypical acoustic pattern in the ASD group could be partially due to the suppression of the F0 range. Moreover, we found that ASD children tended to produce more errors for T2/T2N, T3/T3N than for T1/T1N, T4/T4N. The pattern of tone errors could be explained by the acquisition principle of pitch, similarities among different tones, and tone sandhi. We thus concluded that deficits in pitch processing could be responsible for the atypical tone pattern of ASD children, and speculated that the atypical tonal contours might also be due to imitation deficits. The present findings may eventually help enhance the comprehensive understanding of the representation of atypical pitch patterns in ASD across languages.

## Introduction

Autism spectrum disorder (ASD) is a neurodevelopmental disorder that presents deficits in social interaction, repetitive behaviors, restricted interests, and language (American Psychiatric Association, 2013). The pioneer studies from Kanner (1943) and Asperger (1944) have found that the symptoms of ASD are highly correlated with atypical voice characteristics in speech production, and follow-up research further clarified that exaggerated or robot-like prosody is one of the prominent symptoms of ASD (Pronovost et al., 1966; Ornitz and Ritvo, 1976; Fay and Schuler, 1980; Tager-Flusberg, 1981; Baltaxe and Simmons, 1985, 1992; Paul, 1987). In other words, individuals with ASD are prone to show atypical pitch and pitch variation (Sharda et al., 2010; Kaland et al., 2013; Fusaroli et al., 2017), inappropriate duration and intensity (Bonneh et al., 2011; Scharfstein et al., 2011; Diehl and Paul, 2013), as well as incorrect stress placement (Baltaxe and Guthrie, 1987; Shriberg et al., 2001). Among the abovementioned prosodic features, mean pitch and pitch range are considered to be reliable indicators in distinguishing between speakers with ASD and their typically developing (TD) comparisons through meta-analyses (Fusaroli et al., 2017), as significant differences in pitch are consistently reported in the literature (Sharda et al., 2010; Kaland et al., 2013; Filipe et al., 2014).

From the view of speech production, pitch is how high or low a sound is perceived by the ears and is highly correlated with the physical feature of fundamental frequency (F0), which reflects the frequency of vocal fold vibration (Hirst and Looze, 2021). Thus, the perceived atypical pitch and pitch variation in ASD can be physically measured by F0. However, there is a long-standing debate on whether speakers with ASD exhibit a narrower or a wider F0 range. On the one hand, acoustic analysis in some previous studies indicated that speakers with ASD showed a narrower F0 and more machine-like utterance compared to TD controls (Grossman et al., 2010; Kaland et al., 2013; Nakai et al., 2014). For instance, Nakai et al. (2014) examined Japanese children’s F0 dispersion of words as a measurement of intonation and found smaller F0 variation for Japanese children with ASD compared to their TD peers. On the other hand, some studies found a wider F0 range and higher F0 variation in speakers with ASD who spoke English (Fosnot and Jun, 1999; Hubbard and Trauner, 2007; Diehl et al., 2009). For instance, Diehl et al. (2009) observed the intonation produced by children and adolescents with ASD in a story production task and identified a larger F0 variation for the two ASD groups compared to their corresponding control groups. Kaland et al. (2013) then examined contrastive intonation, produced by TD speakers and speakers with ASD, which could be realized by accentuation and pitch range. They found that while these two groups could produce functionally similar contrastive intonation, the TD group used a larger pitch range compared to the ASD group. According to the above comparisons, it is evident that the F0 patterns in different languages present diversity. All spoken languages use pitch for intonation at the sentence level, whereas in pitch-accent and tone languages, pitch can be used at the word level (Chao, 1933; Cao, 2002; Gussenhoven, 2004). Japanese, as mentioned above, is a pitch-accent language in which pitch is used to indicate word accent. In tonal language, each syllable has its independent tone, which gives pitch an even heavier functional load. A typical example is Mandarin Chinese. In Mandarin Chinese, the four lexical tones which contrast in pitch contour can be realized through F0 modulation (Chen, 2000; Duanmu, 2007). Based on Chao’s five-scale system (Chao, 1968), in which 5 refers to the highest pitch and 1 represents the lowest pitch of a speaker’s pitch range, the four Mandarin lexical tones are denoted as follows. Tone 1 (T1) is a high-level tone (55); tone 2 (T2) is a high-rising tone (35); tone 3 (T3) is a low-dipping tone (213); tone 4 (T4) is a high-falling tone (51). Take the syllable /ma/ as an example. It means “mother, hemp, horse, to scold” when combined with T1, T2, T3, and T4, individually. Thus, the F0 range in Mandarin Chinese may have different patterns from those in the non-tonal languages. Instead of simply debating whether speakers with ASD have a narrower or a wider F0 range, we should also take the function of pitch in the languages they speak into consideration. However, to the best of our knowledge, no studies have depicted the word-level prosodic features in Mandarin-speaking children with ASD. It therefore would be worthwhile to investigate the acquisition of lexical tones in Mandarin-speaking ASD children, and then elaborate the pattern of the atypical pitch in ASD groups to examine whether it is a tonal-language-specific pattern. It would eventually shed light on the understanding of the representation of atypical pitch patterns in ASD across languages.

Previous studies found that toddlers raised in a Mandarin-speaking environment could achieve mastery of Mandarin tones by the age of 3, which was much earlier than their mastery of vowels and consonants (Chao, 1951; Li and Thompson, 1977; Clumeck, 1980; Zhu and Dodd, 2000; Zhu, 2002; Si, 2006). Moreover, the acquisition of different types of tones can be used to reflect the development of children’s speech (Dore, 1975). Li and Thompson (1977) proposed a hierarchy of ease of learning for the four lexical tones: high (T1) > falling (T4) > rising (T2) and dipping (T3). Consistent with the universal acquisition principles of pitch (e.g., Halliday, 1975; Menn, 1976; Crystal, 1979), level and falling tones (i.e., T1 and T4) are easier to acquire than rising tones (i.e., T2). In the meantime, research showed that children who were at an early stage of language development were more likely to use simple contour tones (i.e., rising and falling tones) on simple syllable rimes [i.e., rimes that do not have a diphthong (VV) or a VN sequence; Yang and Lee, 2006]. Although T2 is a simple contour tone, it is easily confused with T3 due to the phonetic similarity between the two tones. Mandarin third tone sandhi, where T3 is converted to a rising tone when followed by another T3, may also lead to the confusion between T2 and T3 (Chao, 1951; Li and Thompson, 1977; Clumeck, 1980; Chen, 2000; Zhu and Dodd, 2000). In addition, rising tones may reflect greater physiological efforts than level and falling tones in production (Ohala and Ewan, 1973; Li and Thompson, 1977). These corroborate the result of previous studies that T1 and T4 were found to be successfully acquired earlier than T2 and T3 (Li and Thompson, 1977; Clumeck, 1980; Zhu, 2002). Compared to T2 and T3, T1 (the level tone), and T4 (the falling tone) are rather easy to be distinguished and produced. Nevertheless, children can still make some errors during the acquisition process by mispronouncing T1 as T4, or vice versa, which may be due to their similar high-pitched onsets (Lee, 2017).

In addition to the four lexical tones, there is also a toneless category, i.e., neutral tone, in Mandarin. The neutral tones in Mandarin serve various lexical and morphosyntactic functions, such as affixation (e.g., /tʰu4 ʦɿ0/ ‘rabbit’), reduplication (e.g., /ma1 ma0/ ‘mom’), and cliticization (e.g., /de0/ ‘s’). Neutral tone is also called the ‘fifth tone’ or T0, and its surface F0 contour is much less stable than that of the full-tone syllables. Unlike lexical tones, early and recent acoustic studies both confirmed a feature of neutral tone that its pitch implementation (i.e., the F0 contour) varied as a function of the preceding tone (e.g., Lin, 1962; Cheng, 1966; Chao, 1968; Wang, 2004; Tang et al., 2019). Specifically, a neutral tone tended to be realized as a mid-falling (41) tone when preceded by a T1, a high-falling (52) tone when preceded by a T2, a mid-level (33) or mid-rising (35) tone when preceded by a T3, and a low-falling (31) tone when preceded by a T4 (Gao, 1980; Wang, 1996). Thus, learning to implement the pitch features of this phonologically under-specified tonal category may pose a challenge for young children, as they must also learn to correctly modify its realization according to the preceding tonal context. According to previous research, neutral tones were mastered much later than lexical tones (Zhu and Dodd, 2000; Gao and Li, 2017; Tang et al., 2019). Children would have developed a phonological category for neutral tone at least by the age of 2–3, but they could not completely achieve an adult-like neutral tone production until 5 years old. Children who have deficits in neutral tone production are prone to substitute the neutral tones with their lexical tone counterparts, and lengthen or omit the neutral tone syllables (Zhu and Dodd, 2000). In addition, the challenge of producing a neutral tone is related to the preceding lexical tone. Tang et al. (2019) reported more off-standard-neutral-tone errors in the disyllabic neutral-tone words initiated by T2 and T3, with a higher onset and wider pitch range in the second syllable.

To gain a better understanding of children’s acquisition of Chinese tone categories, Wu (2021) examined the acoustic properties of tones produced by twelve Mandarin-speaking children with ASD and their TD controls, and utilized a speech repetition task in which participants heard pre-recorded monosyllables and sentences of a short article produced by a female standard Mandarin speaker, and were asked to repeat them. Results showed that the TD children produced the four lexical tones well, while some of the ASD children produced less accurate T3. Specifically, the ASD children tended to incorrectly produce T3 as a falling tone, or as a rising tone, similar to T2. The results also showed larger individual differences for the ASD group, which could be explained by the Intelligence Quotient (IQ) or the mental age difference of the participants. Although some acoustic analysis was conducted, Wu’s study (2021) did not employ statistical analysis to compare tone height and tone shape between the ASD children and the control group. In addition, only monosyllabic words and four lexical tones were taken into consideration in that study.

As mentioned above, tonal-language-speaking children with ASD are more likely to show deficits in lexical tones compared to their TD peers. However, relative to the acquisition of lexical tone studies, few studies acoustically analyzed the production of neutral tone in children with ASD or presented necessary statistical analysis supporting the acoustic observation. Thus, it remains unclear whether Mandarin-speaking children with ASD develop adult-like acoustic realizations of the tone categories (i.e., lexical tones and neutral tone), and how the tones produced by the ASD children differ from those produced by their TD peers. Therefore, in the present study, we attempted to use a repetition pronunciation task, in which participants heard disyllabic words produced by an adult female speaker and were requested to repeat them, to examine the pitch realization of five-to nine-year-olds’ tone production in Mandarin for the following reasons. First, in Mandarin, both intonation and lexical tone involve F0, and they may interact with each other (Chao, 1968; Cao, 2002). Simply, the specific registers of tones are attuned to intonations in Mandarin (Chao, 1968; Cao, 2002). Then, single-word sentences are frequently used in Mandarin. For instance, the word “/ma ma/” (mom) can be used as an interrogative sentence with a rising tone or a declarative sentence with a falling tone. Zheng and Cao (2018) found that intonation can impact the tonal heights and the slopes of contours. Thus, a repetition pronunciation task in which the participants are required to repeat the word they hear can minimize the influence of intonation on tones and eliminate the interference of possibly distinct literacy skills across participants. Second, the repetition task has been a common method used in pronunciation evaluation and rehabilitation training for speakers with ASD (Demouy et al., 2011; Riches et al., 2011). Third, considering that our investigations are not focused on tapping into children’s lexical knowledge (i.e., vocabulary) but on examining their productions of tones through F0 modulation, a repetition task is considered to be suitable for the purpose of the present study. Fourth, the task was not too challenging for the ASD children and they could fully understand the procedure of the experiment. Besides, this age range from five to nine was selected since previous studies had reported that by the age of five, children will have had the ability to produce adult-like neutral tone contours across all tonal contexts (Tang et al., 2019). Moreover, although symptoms of ASD usually manifest within the first 3 years of life (Montiel-Nava et al., 2017; Sheldrick et al., 2017), the majority of children with ASD are generally not diagnosed until the age of five (Filipek et al., 1999).

Taken together, we hypothesized that although lexical tones are relatively easy to acquire for Mandarin-speaking children, the F0 realization of different lexical tones may vary between the ASD and TD groups. Moreover, Mandarin-speaking children with ASD may also have difficulties in producing contextually conditioned neutral tones in an adult-like way. Thus, comparisons of how Mandarin tones are produced by children with ASD and their TD peers will help elaborate the atypicality of pitch patterns in ASD children, and then achieve a comprehensive understanding of the representation of atypical pitch patterns in ASD across languages. Moreover, the current findings of pitch patterns in Mandarin-speaking children may provide the cues about prosodic atypicality in the clinical diagnosis of ASD.

## Materials and methods

### Participants

A total of forty children were included in this study, with 20 ASD children (8 girls) as the experimental group and 20 age-matched TD children (10 girls) as the control group. The children with ASD were recruited from the Cangzhou Research Centre for Child Language Rehabilitation. The clinical diagnosis of ASD was established according to the Diagnostic and Statistical Manual of Mental Disorders (5th ed.; DSM-5; American Psychiatric Association, 2013) criteria for ASD and further confirmed using the Autism Diagnostic Observation Schedule, Second Edition (ADOS-2; Lord et al., 2012) by pediatricians and child psychiatrists in child hospitals. The ADOS-2 is a standardized and semi-structured assessment instrument, allowing examiners to observe and evaluate the ASD defining symptoms in the course of structured playful and interview-based interactions. The ADOS-2 consists of five different modules suited for children with different levels of language development, and also provides instructions for calculating the ADOS-2 Comparison Score on a scale of 1–10 (10 representing the highest severity of autism-related symptoms) to gauge autism severity. In the present study, the ADOS-2 Module 2 was mainly used for diagnosis according to the language level reached by the recruited children. The range of ADOS-2 scores was from 6 to 9, with Comparison Score 6 for two children, Comparison Score 7 for eleven children, Comparison Score 8 for six children, and Comparison Score 9 for one child. The age-matched TD children were then recruited from a local kindergarten with age-matched neurotypical speech, language, and cognitive levels. Although the TD children were not specially screened for autism symptoms *via* standardized instruments, they met none of the DSM-5 criteria for ASD from an interview with their parents or teachers. As presented in Table 1, the children with ASD did show significant speech, language, and cognitive delays compared to age-matched TD children. All these ASD Children have been receiving Mandarin education in school and are rarely influenced by the local dialects. During the recruiting phase, we actually tested the children’s Mandarin proficiency to ensure that they were fully capable of speaking Mandarin. We also made sure that all the participants were free from interference from any other dialects or languages. The ASD participants presented speech production deficits and do not have cerebral palsy or tuberous sclerosis, hearing/sight impairment, Down syndrome, uncontrolled seizures, and organic impairment of oral or laryngeal structures (Wan et al., 2011; Chenausky et al., 2016). Through the assessments, all the recruited children with ASD were confirmed to be eligible for the study. The experimental protocol for the study was obtained from the Ethical Committee of School of Psychological and Cognitive Sciences at Cangzhou Normal University, Hebei, China. Written informed consent was signed by all the children’s parents before the assessments and the experiment.

**Table 1:** Characteristics of children with autism spectrum disorder (ASD) and age-matched typically developing (TD) children.

Variable	ASD (*n* = 20)		TD (*n* = 20)		*t*	*p*
	M	SD	M	SD		
Age (years)	6.95	1.47	6.55	1.43	0.87233	0.389
Language ability	48.5	23.73	89.1	8.69	−7.1858	<0.001
Nonverbal IQ (Standard scores)	7.05	5.78	14.6	3.91	−7.4455	<0.001
Working memory	67.29	14.76	101.3	15.96	−4.8372	<0.001

### Materials

A list of self-compiled materials was developed for the pronunciation task. The list was composed of four sets of reduplicated nouns, with the base morpheme of each set carrying T1, T2, T3, or T4, respectively. Due to the reduplication process, the tone of the second syllable was realized as a neutral tone, allowing us to examine the F0 realization of the four neutral tones (T1N, T2N, T3N, T4N) preceded by the four lexical tones. In addition, half-third sandhi was applied when the base morpheme carried a T3, in which case, the first low-dipping T3 became a low-falling tone. Each set contained 5 reduplicated nouns, resulting in a total of 20 target stimuli for the experiment. All materials were recorded uniformly by a professional female broadcaster using the software Praat (Boersma and Weenink, 2022), with a sampling rate of 44,100 Hz, 16-bit mono *via* the TAKSTAR PCM-5520 professional condenser microphone. The materials adopted in this study were listed in Table 2.

**Table 2:** Reduplication stimuli in this study.

T1 + T1N	T2 + T2N	T3 + T3N	T4 + T4N
妈妈 ‘mom’	爷爷 ‘grandpa’	奶奶 ‘grandmother’	爸爸 ‘dad’
叔叔 ‘uncle, father’s younger brother’	伯伯 ‘uncle, father’s older brother’	宝宝 ‘baby’	弟弟 ‘younger brother’
哥哥 ‘older brother’	婆婆 ‘mother-in-law, grandma’	姐姐 ‘older sister’	妹妹 ‘younger sister’
星星 ‘star’	娃娃 ‘doll’	粑粑 ‘feces’	泡泡 ‘bubble’
车车 ‘car’	糖糖 ‘candy’	狗狗 ‘doggy’	豆豆 ‘bean’

### Procedure

Before the pronunciation experiment, all the children were assessed on their language ability, nonverbal IQ, and working memory capacity. First, their language ability was evaluated through five subtests, including the Test of Mandarin Grammar, Word Definition Test, Rapid Automatized Naming, Narrative Test, and Sentence Comprehension Test (Ning, 2013). These tests examined the children’s phonological, lexical, syntactic, and semantic abilities, and lasted approximately 30 min. Then, the children’s nonverbal IQ was assessed with the Primary Test of Nonverbal Intelligence, a research-based method designed to assess reasoning abilities in young children aged 3; 0–9; 11 (PTONI; Ehrler and McGhee, 2008), which took around 15 min. Then, the children’s working memory capacity was assessed with a forward digit span test adapted from Yan et al. (2021)’s paradigm, in which the participants heard a series of numbers and were asked to recall them immediately. Each series of numbers were divided into two chunks, with one chunk containing two to nine digits. The response of each chunk was counted as correct and awarded 0.5 points only when the participants could correctly recall every digit in the right order. The full score of the forwarding digit span test was 8.

**Figure 1:**
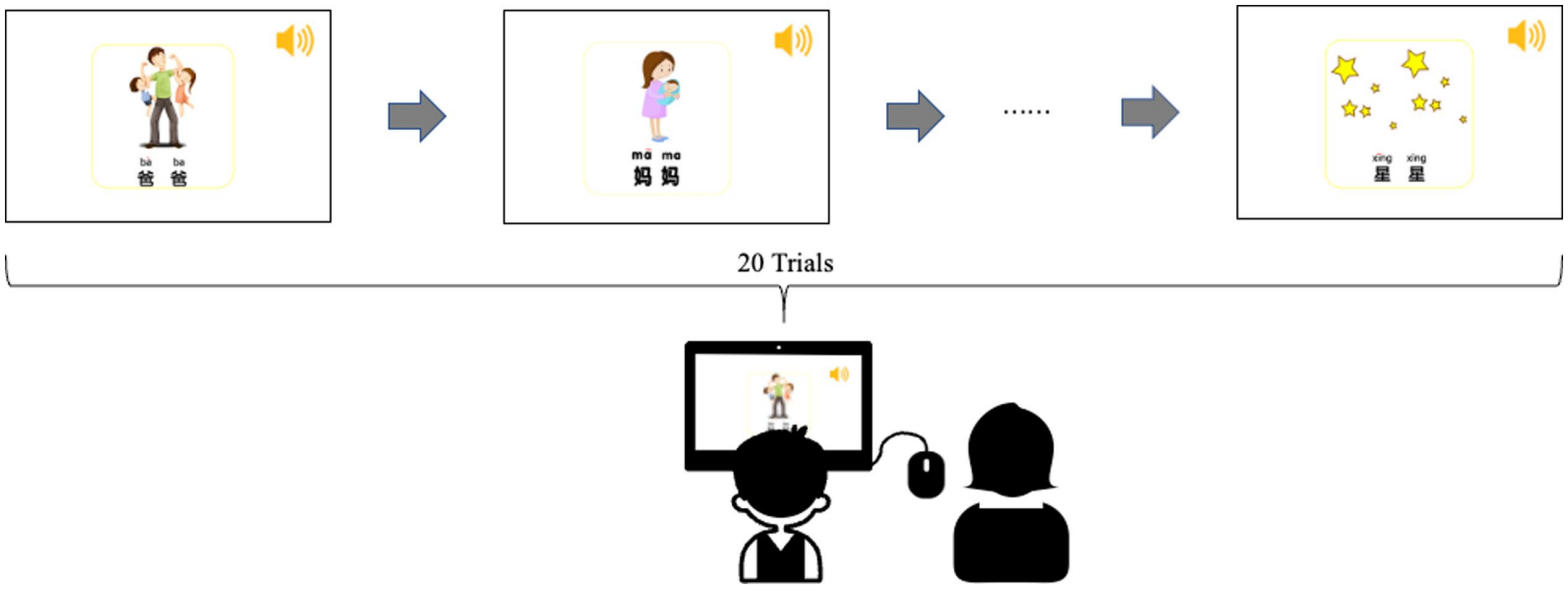
The experimental paradigm. During the task, the children were asked to pronounce the reduplicated target words on the slides following the audio materials. The experimenter controlled the playback mode of each slide, which was set to the mouse click.

In the pronunciation experiment, the 20 reduplicated nouns were presented using PowerPoint, with one word on each slide. As shown in Figure 1, each reduplicated noun was presented using Chinese characters with pinyin (the Chinese spelling system) sitting above them. In addition, each reduplicated noun was accompanied by an image to make the experiment more appealing to the children. During a trial, the target word, the image, and the audio material would be presented to the children all at once. As soon as the audio was over, the participants were asked to pronounce the target word twice. They were also encouraged to pronounce it more than twice if they wanted. Their pronunciations were recorded by the experimenter. On occasions when the children did not respond or his/her pronunciation did not match the target word, the experimenter played the next slide by clicking the mouse. There were 20 trials in total and presented in a fully-randomized order. After the recording, the best two tokens of every target word were selected, resulting in 1600 disyllabic tokens (40 children × 20 reduplicated nouns × 2 repetitions). Then, a pre-screening process was conducted in which the tokens that were produced too loudly, too lowly, or unclearly caused by the appropriate distance from the microphone were excluded. Finally, 798 (99.75%) disyllabic tokens for the TD group and 758 (94.75%) disyllabic tokens for the ASD group were included and subject to further analyses. It took about 3 min to complete the pronunciation experiment.

### Data analysis

Before acoustic analysis, the 1,556 disyllabic productions were scrutinized by the third and fourth authors of this study, who are phonetically trained native speakers of Standard Mandarin. The first and second syllables of the disyllabic productions were evaluated separately and classified into one of the following categories. First, both the segments and the tone are correct. Second, the segments are incorrect but the tone is correct. Third, the segments are correct but the tone is incorrect. Fourth, neither segments nor tone is correct. Since the current study focuses on Mandarin tone production, syllables that fell into the first two categories were subject to tone analyses (i.e., acoustic and growth curve analyses) and coded as 1, whereas syllables classified into the last two categories were subject to error analysis and coded as 0. Tones which had the following patterns were judged as errors. (1) For the lexical tones, T1 was not perceived as a high-level tone; T2, not as a rising tone; T3, not as a low-falling tone due to the half-third sandhi; T4, not as a high-falling tone. (2) For the neutral tone, it was not perceived with a short duration with reference to the preceding lexical tone syllable, or not perceived as a correct pitch, i.e., T1N/T2N/T4N, not as a falling pitch, T3N, not as a mid-level or mid-rising pitch. The Kappa Statistic was conducted on the two raters’ ratings (1, 0) to measure inter-rater reliability using IBM SPSS Statistics for Windows, Version 22.0. Results showed that the inter-rater reliability was found to be Kappa = 0.723 (*p* < 0.001), indicating that there was substantial agreement between the two raters. For the TD group, 2 tokens (0.25%) of the first syllable and 62 tokens (7.77%) of the second syllable were excluded from the tone analyses, while for the ASD group, 158 tokens (20.84%) of the first syllable and 144 tokens (19%) of the second syllable were excluded from tone analyses. These tokens, which had incorrectly produced tones, were subject to tone error analysis. Lastly, the two raters did not report any creakiness in the productions of both groups of children.

The F0 of the target words was measured using the software Praat (Boersma and Weenink, 2022). For the 18 morphemes that begin with a voiceless stop, voiceless affricate, voiceless fricative, or nasal, F0 of the first syllable was measured from the onset of vocal fold vibration (the onset of periodicity in the waveform) to the onset of the initial consonant of the second syllable. For the F0 of the second syllable, it was measured from the onset of vocal fold vibration to the point at which the second formant disappeared in the spectrogram. Regarding [je2 je2] “grandfather” and [wa2 wa2] “doll” which begin with a glide, F0 of the first syllable was measured from the onset of vocal fold vibration to the highest point of F0 in the pitch analysis in Praat (Shi, 2013), while the F0 of the second syllable was measured from the highest point to the point at which the second formant disappeared in the spectrogram. F0 height and contour of both syllables were analyzed using Xu (2013) in Praat. Time-normalized F0 was generated by measuring the F0 value of every 11.11% of the tone, leading to ten F0 measurements for each syllable.

After time-normalized F0 measurements were yielded, the F0 values were then converted to semi-tone using the formula in (1) below to approximate pitch perception (Rietveld and Chen, 2006). Next, the semi-tone values were transformed into z-scores using the formula in (2) below for all measurements of a given speaker to minimize individual and gender differences in F0 (Rose, 1987; Zhu, 2004). Finally, semi-tone F0 z-scores were analyzed using growth curve analysis for tone height and shape (Mirman et al., 2008; Mirman, 2014; Chien and Jongman, 2019; Tu and Chien, 2022).


(1) ST=39.87×log(Hz50)

(2) $$zSTx=\frac{STx-\frac{1}{n}\sum_{i=1}^{n}STi}{\sqrt{\frac{1}{n-1}\sum_{i=1}^{n}{\left(STi-\frac{1}{n}\sum_{i=1}^{n}STi\right)}^{2}}}$$

## Results

### Lexical tones

The semi-tone z-scores of lexical tones (i.e., T1, T2, T3, and T4) were modeled using growth curve analysis (Mirman et al., 2008; Mirman, 2014; Chien and Jongman, 2019; Tu and Chien, 2022), which allowed us to model the curvilinear relationship between the four lexical tones and the normalized time and the contour of participants’ overall tonal production curve instead of their average tonal values (e.g., F0, semi-tone z-scores) in arbitrarily selected time windows.

A series of growth curve analyses were conducted on the semi-tone z-scores of the four lexical tones using the lme4 package in R (Bates et al., 2015), with *p*-values calculated by the lmerTest package (Kuznetsova et al., 2017). Time (linear, quadratic, cubic, i.e., ot1, ot2, ot3), Group (ASD, TD), Tone (T1, T2, T3, T4), and their interactions were treated as fixed factors. Three separate models containing only the linear time term, the linear and quadratic time terms, and all three time terms were first run and compared to determine whether subsequent models should include only one, or two, or all three time polynomials. Results of likelihood ratio tests showed that the model with all three time polynomials was the best one (*χ^2^*(9) = 38.787, *p* < 0.001). Therefore, subsequent models with Group and Tone as additional fixed factors were built on this model. For Group, the ASD group was entered as the baseline to which the TD group was compared. For Tone, T1 was treated as the baseline to which the other three lexical tones were compared. All models also contained a set of random effects to capture participant-level and group-level variability in all three time polynomials. Likelihood ratio tests were employed by using a forward-fitting method to determine the model that could account for significantly more variance of the data than all simpler models. The model containing the following fixed factors and random effect structure was determined as the optimal model: (ot1 + ot2 + ot3) * Group * Tone + (ot1 + ot2 + ot3|Participant) + (ot1 + ot2 + ot3|Participant: Group) (*χ^2^*(16) = 173.204, *p* < 0.001).

Since the best model consisted of interaction effects among Group, Tone, and Time, suggesting that the patterns of lexical tones differed significantly between the two groups, a series of growth curve analyses within each lexical tone was conducted to further evaluate whether the height and shape of each tone differed between the two groups of participants. For each series of analyses, three Time-only models were first built, with the three time polynomials being added one at a time. Then likelihood ratio tests were conducted to determine the best model with the most appropriate time polynomials on which subsequent models were built. These subsequent models contained Time, Group (ASD, TD), and their interactions as fixed factors. For Group, the ASD group was entered as the baseline to which the TD group was compared. A set of random effects were also used in all models to capture participant-level and group-level variability in their corresponding time polynomials. Likelihood ratio tests were employed by using a forward-fitting method to determine the optimal model for each series of analyses.

In the present study, to demonstrate the significant difference between the tones (T1, T2, T3, and T4) produced by the children with ASD and those by the TD controls was due to ASD, the growth curve analysis should reveal either the effect of Group (ASD group vs. TD group) or interactions between Group and at least one of the time polynomials. The Group effect would suggest differences in tone height and the interaction effects would indicate differences in tone shape between the two groups. Only the results of the best model for each tone are presented below.

**Figure 2:**
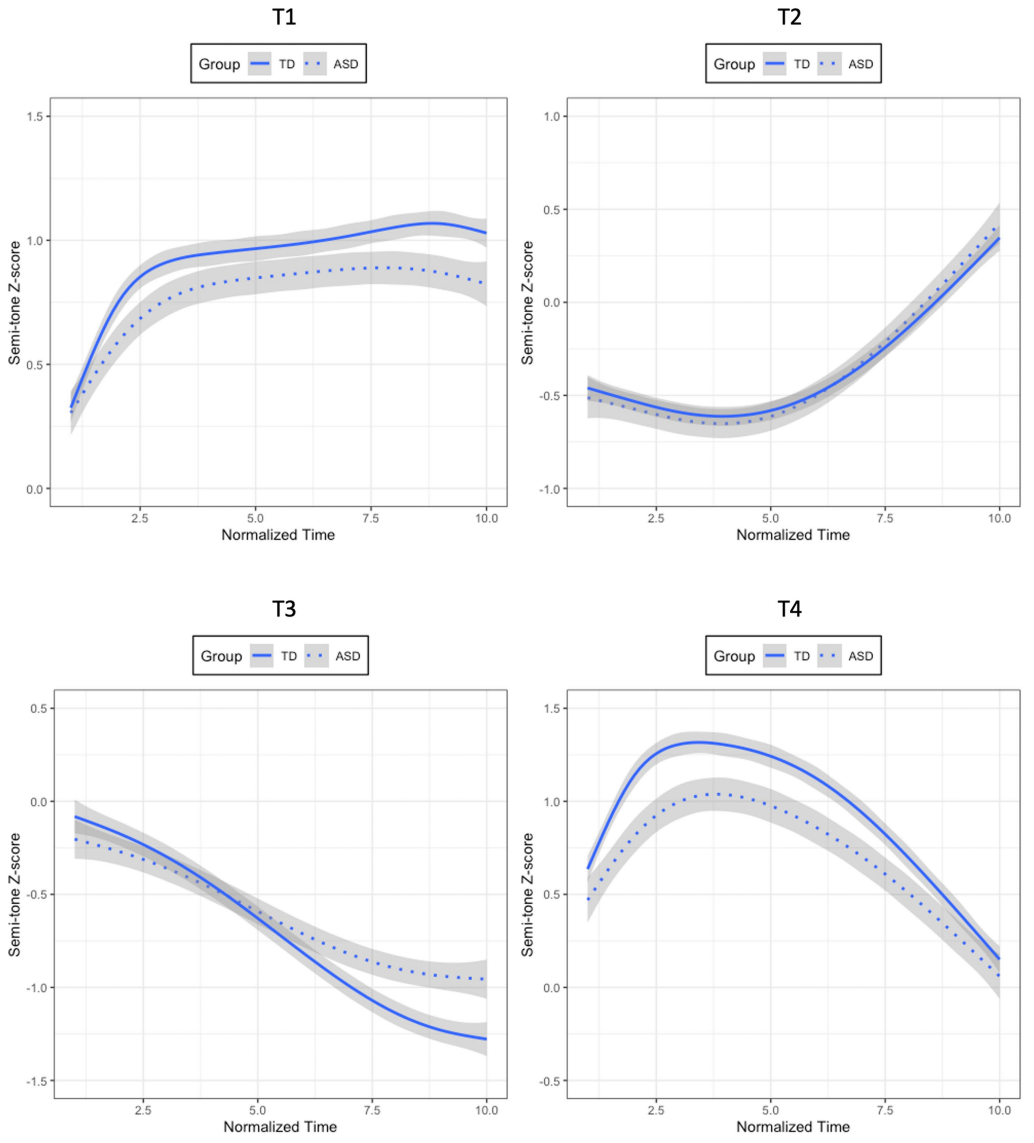
Mean semi-tone z-score tracks of T1 (top-left panel), T2 (top-right panel), T3 (bottom-left panel), and T4 (bottom-right panel) for the ASD and TD children.

For T1, the model with Time and Group as fixed factors was marginally significantly better than the one without Group (*χ^2^*(1) = 3.263, *p* = 0.071). The model containing Time, Group, and their interactions as fixed factors was not significantly better than the one without interactions (*χ^2^*(3) = 3.882, *p* = 0.274). Therefore, the model containing Time and Group as fixed factors was determined as the optimal model (Semi-tone z-scores ~ (ot1 + ot2 + ot3) + Group + (ot1 + ot2 + ot3|Participant) + (ot1 + ot2 + ot3|Participant: Group)). The results of this model showed that T1 produced by the ASD children was marginally significantly different from that produced by the TD children in tone height, with the T1 produced by the former being lower than that produced by the latter (Group: *β* = 0.178, *SE* = 0.092, *t* = 1.928, *p* = 0.062). Since Group did not interact with any of the time polynomials, T1 produced by the two groups of participants did not differ in tone shape, as shown in the top-left panel of Figure 2.

Regarding T2, the model including Time and Group as fixed factors failed to explain significantly more variance of the data than the one with only Time (*χ^2^*(1) = 0.348, *p* = 0.555). Neither did the model containing Time, Group, and their interactions account for significantly more variance of the data than the one with only Time and Group (*χ*^2^(3) = 0.869, *p* = 0.719). Thus, the results of T2 demonstrated that T2 produced by the ASD group and that produced by the TD group did not significantly differ from each other in tone height or tone shape, as shown in the top-right panel of Figure 2.

In terms of T3, the model containing Time, Group, and their interactions was marginally better than the one without interactions (*χ^2^*(3) = 7.611, *p* = 0.055). Therefore, it was selected as the best model (Semi-tone z-scores ~ (ot1 + ot2 + ot3) * Group + (ot1 + ot2 + ot3|Participant) + (ot1 + ot2 + ot3|Participant: Group)). The results of this model revealed that T3 produced by the ASD children was not significantly different from that produced by the TD controls in tone height (Group: *β* = −0.056, *SE* = 0.101, *t* = −0.550, *p* = 0.586), while these two groups produced significantly different T3 contours (Linear × Group: *β* = −0.480, *SE* = 0.170, *t* = −2.827, *p* = 0.007). More specifically, the negative coefficient estimate for the interaction between the linear time term and Group suggested that T3 produced by the ASD group had a less negative slope relative to that produced by the TD group, as shown in the bottom-left panel of Figure 2.

For T4, the model with Time and Group as fixed variables accounted for significantly more variance of the data than the one without Group (*χ^2^*(1) = 5.658, *p* = 0.017). The model including Time, Group, and their interactions as fixed variables failed to explain significantly more variance than the one without interactions (*χ^2^*(3) = 3.576, *p* = 0.311). Hence, the model with Time and Group was determined as the optimal model (Semi-tone z-scores ~ (ot1 + ot2 + ot3) + Group + (ot1 + ot2 + ot3|Participant) + (ot1 + ot2 + ot3|Participant: Group)). The results of this model showed a significant effect of Group (*β* = 0.187, *SE* = 0.072, *t* = 2.588, *p* = 0.015). Furthermore, the positive coefficient estimate indicated that the T4 produced by the ASD group was lower than that produced by the TD group, as illustrated in the bottom-right panel of Figure 2. The results of the four lexical tones are summarized in Table 3 below.

**Table 3:** Summary results of lexical tone height and shape between the ASD and TD groups.

Pattern	Tone height	Tone shape
Tone		
Tone 1	ASD with a lower tone height	No difference
Tone 2	No difference	No difference
Tone 3	No difference	ASD with a less negative tone slope
Tone 4	ASD with a lower tone height	No difference

**Figure 3:**
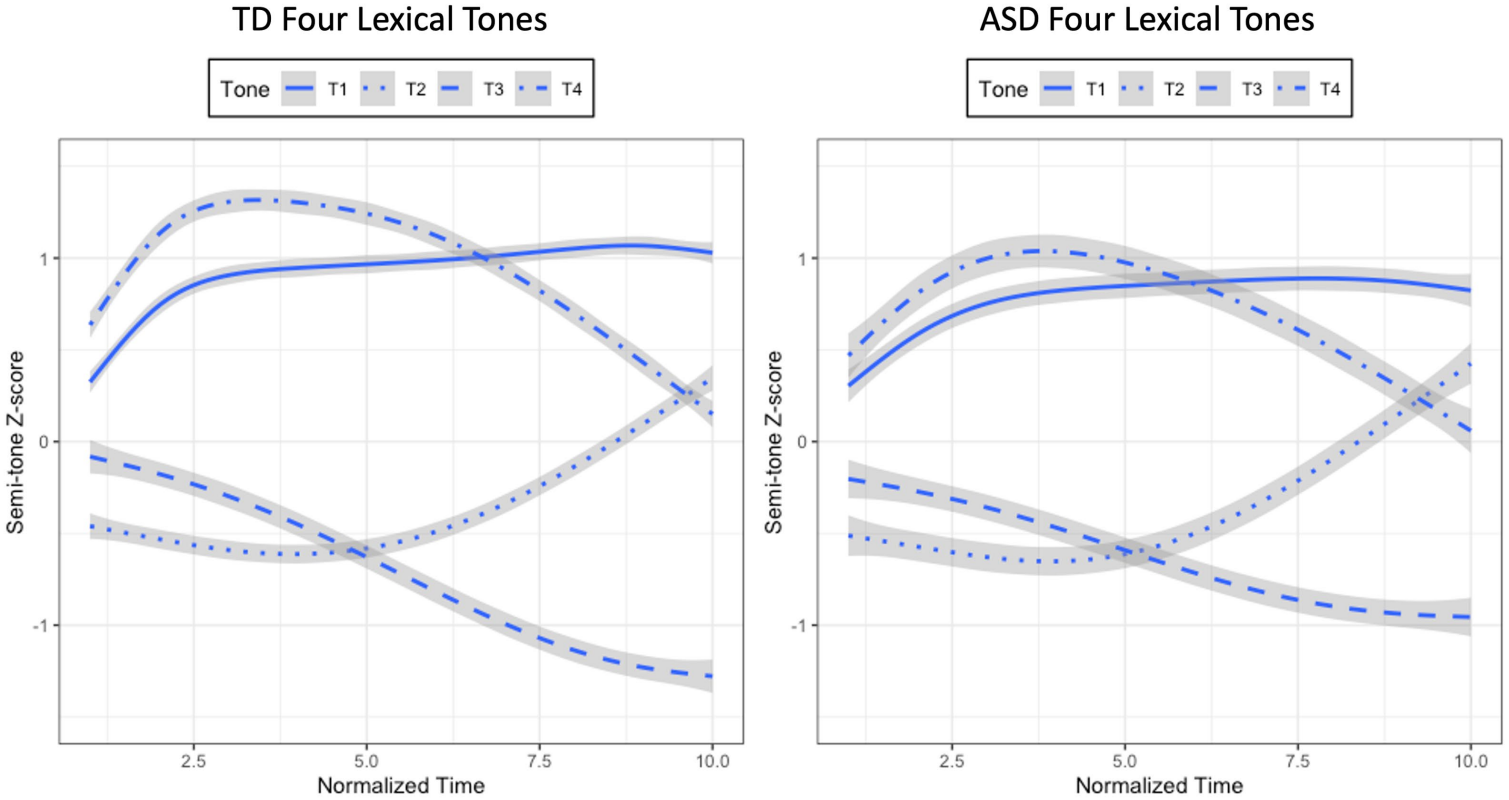
Mean semi-tone z-score tracks of four lexical tones for the TD (left panel) and ASD (right panel) children.

Given that the model containing the interactions between Time, Group, and Tone was the best, two sets of growth curve analyses were conducted within the TD group and the ASD group to evaluate whether the four lexical tones within each group were significantly different in tonal contours. If Tone interacted with any of the time polynomials, it would suggest that the four lexical tones were statistically distinct from one another. For each series of analyses, three Time-only models were first built, with the three time polynomials being added one at a time. Then likelihood ratio tests were conducted to determine the best model with the most appropriate time polynomials on which subsequent models were built. These subsequent models contained Time, Tone (T1, T2, T3, T4), and their interactions as fixed factors. For Tone, T1 was treated as the baseline to which the other tones were compared. A set of random effects were also used in all models to capture participant-level and tone-level variability in their corresponding time polynomials. Likelihood ratio tests were employed by using a forward-fitting method to determine the optimal model for each series of analyses. For the TD group, the model containing Time, Tone, and their interactions was significantly better than the one without interactions, thus, determined as the best model (*χ*^2^(9) = 2321.1, *p* < 0.001; SF0Z ~ (ot1 + ot2 + ot3) * Tone + (ot1 + ot2 + ot3|Participant) + (ot1 + ot2 + ot3|Participant: Group)). For the ASD group, the model with Time, Tone, and their interactions significantly improved the model without interactions, hence, determined as the optimal model (*χ*^2^(9) = 855.3, *p* < 0.001; SF0Z ~ (ot1 + ot2 + ot3) * Tone + (ot1 + ot2 + ot3|Participant) + (ot1 + ot2 + ot3|Participant: Group)). The interaction effects indicated that the four lexical tones produced by both TD and ASD children were distinct in tonal contours, as shown in Figure 3.

Taken together, Figure 3 shows that the four lexical tones produced by the ASD and TD groups were consistent with those described in the literature: T1 as high-level, T2 as high-rising, T3 as low-falling after undergoing half-third sandhi, and T4 as high-falling. Although T2 produced by the two groups did not differ from each other statistically, the growth curve analyses revealed differences in tone height for T1 and T4, and differences in tone shape for T3 between the two groups. Interestingly, when comparing all the four lexical tones together produced by the two groups, it seems that the four lexical tones were not as distinct from one another for the ASD group (i.e., closer to one another in tone space), as revealed by the lower tone height for T1, the lower tone height of T4, and the shallower slope for T3. These results are subject to discussion in Section 4.

### Neutral tones

A series of growth curve analyses were conducted on the semi-tone z-scores of the four neutral tones following T1, T2, T3, and T4 (henceforth, T1N, T2N, T3N, and T4N) to model their height and shape using the lme4 package in R (Bates et al., 2015), with *p*-values calculated by the lmerTest package (Kuznetsova et al., 2017). Time (linear, quadratic, cubic, i.e., ot1, ot2, ot3), Group (ASD, TD), Tone (T1N, T2N, T3N, T4N), and their interactions were set as fixed factors. Three individual models with only the linear time term, the linear and quadratic time terms, and all three time terms were first run and compared to determine whether subsequent models should include only one, or two, or all three time polynomials. Results of likelihood ratio tests showed that the model with all time polynomials was the optimal one (*χ^2^*(9) = 23.710, *p* = 0.005). Therefore, subsequent models with Group and Tone as additional fixed factors were built on this model. For Group, the ASD group was treated as the baseline to which the TD group was compared. For Tone, T1N was entered as the baseline to which the other three neutral tones were compared. All models also included a set of random effects to capture participant-level and group-level variability in their corresponding time polynomials. Likelihood ratio tests were employed by using a forward-fitting method to determine the model that could account for significantly more variance of the data than all simpler models. The model containing the following fixed factors and random effect structure was determined as the best model: (ot1 + ot2 + ot3) * Group * Tone + (ot1 + ot2 + ot3|Participant) + (ot1 + ot2 + ot3|Participant: Group) (*χ*^2^(16) = 76.826, *p* < 0.001).

Given that the optimal model contained interaction effects between Group, Tone, and Time, which indicated that the patterns of neutral tones differed significantly between the two groups, a series of growth curve analyses was conducted within each neutral tone to further examine whether individual neutral tones produced by the ASD and TD children were significantly different. For each series of analyses, three Time-only models were first built, with the three time polynomials being included one at a time. Likelihood ratio tests were then conducted to determine the best model with the most appropriate time polynomials on which subsequent models were built. These subsequent models included Time, Group (ASD, TD), and their interactions as fixed factors. For Group, the ASD group was entered as the baseline to which the TD group was compared. A set of random effects were also entered in all models to capture participant-level and group-level variability in their corresponding time polynomials. Likelihood ratio tests using a forward fitting method were employed to determine the model that could explain significantly more variance of the data than all the other simpler models. Results of the best model for each series of analyses are presented below.

**Figure 4:**
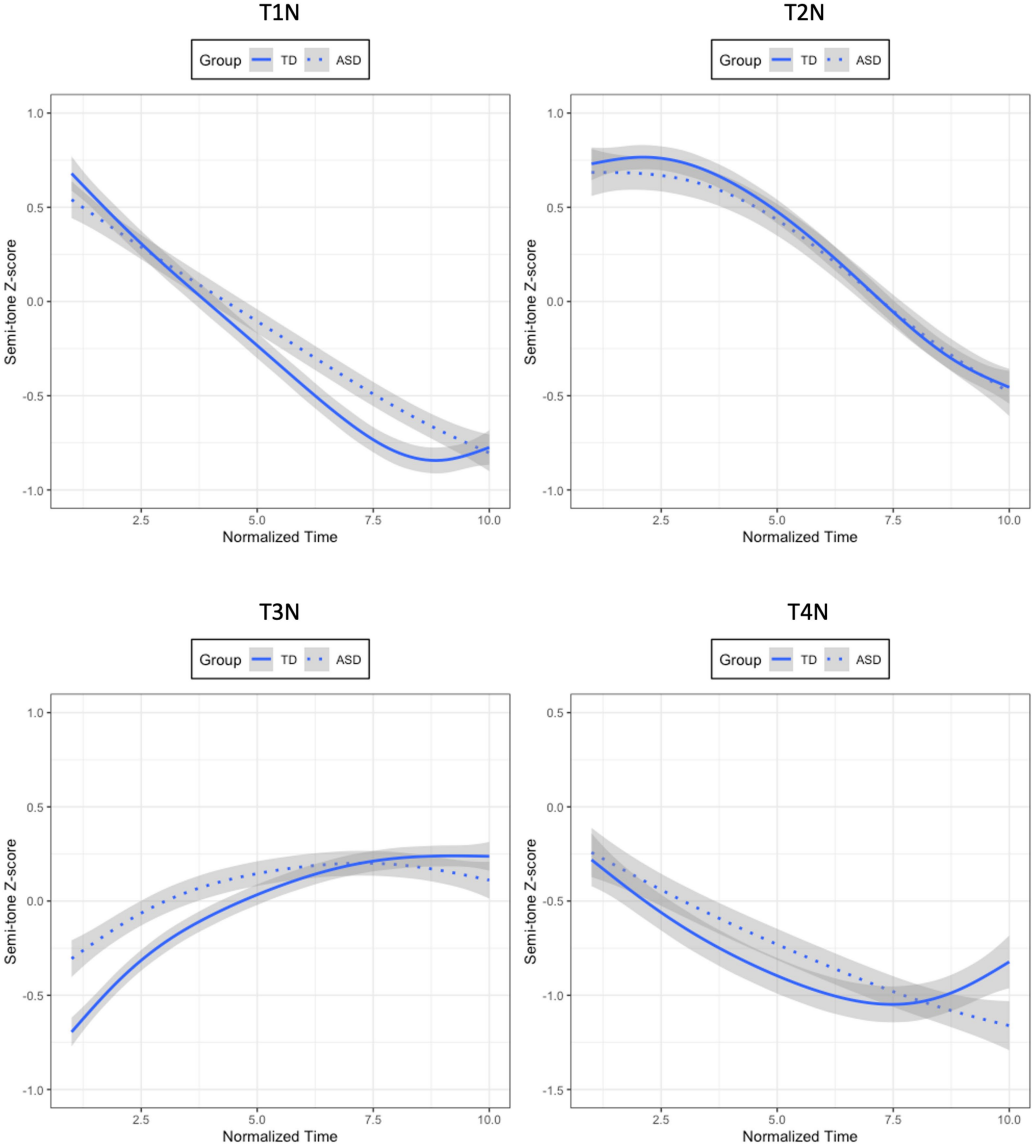
Mean semi-tone z-score tracks of T1N (top-left panel), T2N (top-right panel), T3N (bottom-left panel), and T4N (bottom-right panel) for the ASD and TD children.

For T1N, the model containing Time, Group, and their interactions was determined as the best model (Semi-tone z-scores ~ (ot1 + ot2) * Group + (ot1 + ot2 |Participant) + (ot1 + ot2|Participant: Group); *χ^2^*(2) = 10.613, *p* = 0.005). For this model, Group did not reach significance (*β* = −0.087, *SE* = 0.083, *t* = −1.043, *p* = 0.303), suggesting that T1N did not differ in height between the two groups. However, the interaction between the quadratic time term and Group was significant, indicating that the shape of T1N produced by the ASD and TD groups was significantly different (*β* = 0.283, *SE* = 0.131, *t* = 2.167, *p* = 0.036). More specifically, the positive coefficient estimate of this interaction suggested that T1N had a less convex shape (U shape) for the ASD group than for the TD group, as in the top-left panel of Figure 4.

In terms of T2N, the model with Time and Group was not significantly better than the one with only Time (*χ*^2^(1) = 0.429, *p* = 0.512). Neither was the model containing Time, Group, and their interactions significantly better than the one without interactions (*χ^2^*(3) = 1.128, *p* = 0.770). The results showed that T2N was very similar in tone height and tone shape between the two groups, as in the top-right panel of Figure 4.

For T3N, the model containing Time, Group, and their interactions could account for significantly more variance of the data than the one without interactions (Semi-tone z-scores ~ (ot1 + ot2) * Group + (ot1 + ot2 |Participant) + (ot1 + ot2|Participant: Group); *χ^2^*(2) = 9.276, *p* = 0.010), hence, determined as the optimal model. The results of this model showed that Group was not significant, indicating that the ASD and TD groups produced similar T3N in tone height (*β* = −0.089, *SE* = 0.096, *t* = −0.923, *p* = 0.362), while the interaction between the linear time polynomial and Group was significant, demonstrating that the two groups of participants produced significantly different T3N in tone shape (*β* = 0.480, *SE* = 0.157, *t* = 3.055, *p* = 0.004). More specifically, the positive coefficient estimate suggested that the T3N produced by the ASD group had a less positive slope than that produced by the TD group, as shown in the bottom-right panel of Figure 4.

Regarding T4N, the model with Time, Group, and their interactions could explain significantly more variance of the data than the one without interactions (Semi-tone z-scores ~ (ot1 + ot2) * Group + (ot1 + ot2|Participant) + (ot1 + ot2|Participant: Group); *χ^2^* (2) = 7.127, *p* = 0.028), thus, determined as the best model. The results of this model revealed that Group did not reach significance (*β* = −0.204, *SE* = 0.167, *t* = −1.221, *p* = 0.230), indicating that the T4N produced by the ASD children and that produced by the TD controls did not differ significantly in tone height. In addition, the interaction between the quadratic time polynomial and Group was significant (*β* = 0.433, *SE* = 0.159, *t* = 2.723, *p* = 0.010), suggesting that the shape of T4N was different between the two groups. More specifically, the positive coefficient estimate demonstrated that the T4N produced by the ASD children had a less convex shape (U shape) than that produced by the TD children. The results of the four neutral tones are summarized in Table 4 below.

**Table 4:** Summary results of neutral tone height and shape between the ASD and TD groups.

Pattern	Tone height	Tone shape
Tone		
T1N	No difference	ASD with a less convex tone shape
T2N	No difference	No difference
T3N	No difference	ASD with a less positive tone slope
T4N	No difference	ASD with a less convex tone shape

**Figure 5:**
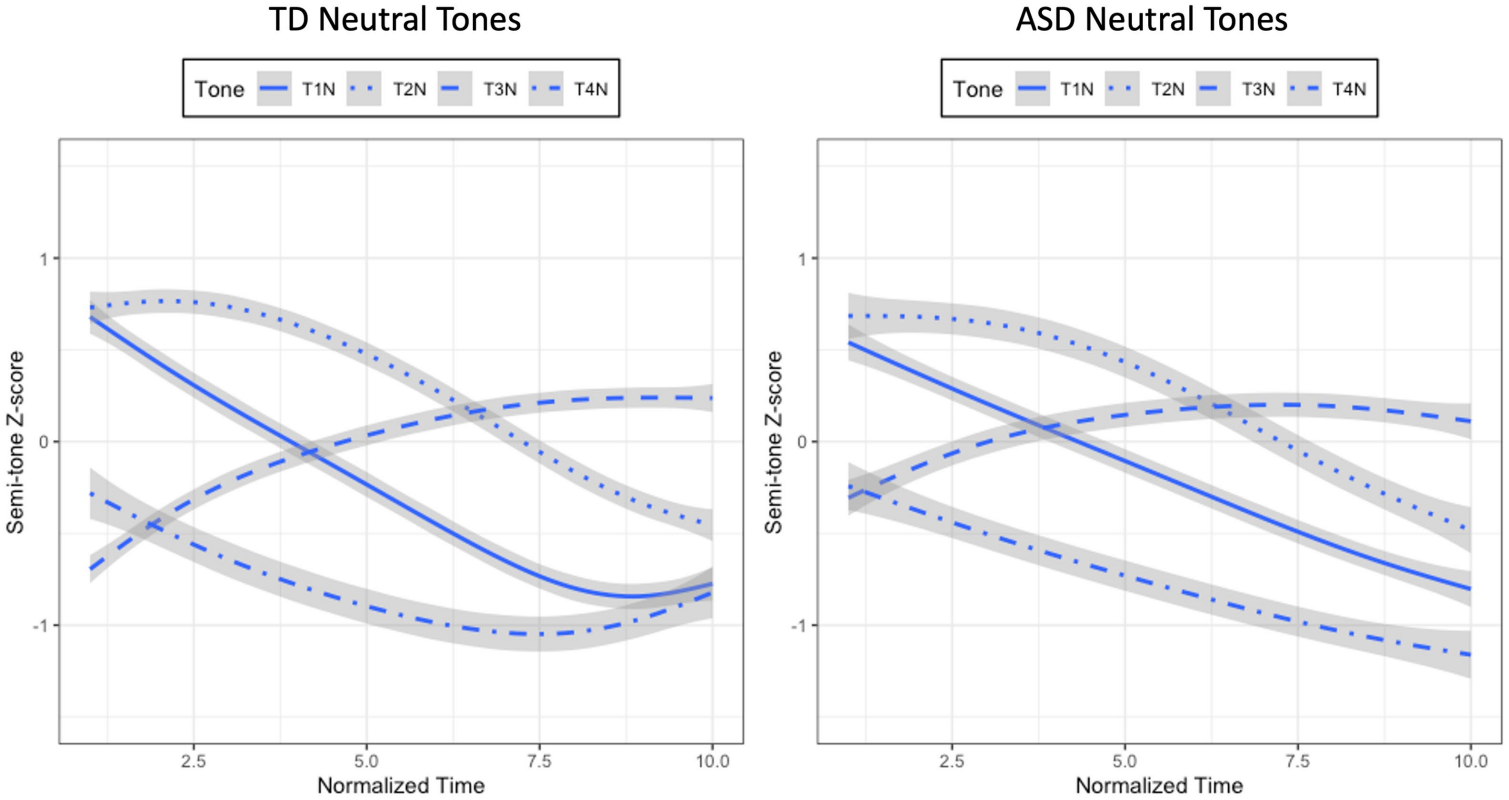
Mean semi-tone z-score tracks of four neutral tones for the TD (left panel) and ASD (right panel) children.

Since the model having the interactions between Time, Group, and Tone was the best, two additional series of growth curve analyses were run within the TD group and the ASD group to examine whether the four neutral tones within each group were significantly different in tonal contours. If Tone interacted with any of the time polynomials, it would indicate that the four neutral tones were statistically distinct from one another. For each series of analyses, three Time-only models were first constructed, with the three time polynomials being included one at a time. Then likelihood ratio tests were conducted to determine the best model with the most appropriate time polynomials on which subsequent models were built. These subsequent models contained Time, Tone (T1N, T2N, T3N, T4N), and their interactions as fixed factors. For Tone, T1N was treated as the baseline to which the other tones were compared. A set of random effects were also used in all models to capture participant-level and tone-level variability in their corresponding time polynomials. Likelihood ratio tests were employed by using a forward-fitting method to determine the optimal model for each series of analyses. For the TD group, the model containing Time, Tone, and their interactions was significantly better than the one without interactions. Hence, it was considered the best model (*χ^2^*(9) = 1,047, *p* < 0.001; SF0Z ~ (ot1 + ot2 + ot3) * Tone + (ot1 + ot2 + ot3|Participant) + (ot1 + ot2 + ot3| Participant: Group)). For the ASD group, the model with Time, Tone, and their interactions significantly improved the model without interactions. Therefore, it was deemed the optimal model (*χ^2^*(6) = 423.22, *p* < 0.001; SF0Z ~ (ot1 + ot2) * Tone + (ot1 + ot2|Participant) + (ot1 + ot2|Participant: Group)). These interaction effects suggested that the four neutral tones produced by both groups of children were statistically dissimilar in tonal contours, as shown in Figure 5.

In summary, the four neutral tones produced by the ASD children and their TD controls exhibited typical neutral tone contours as described in previous studies, with the neutral tone preceded by T1 and T2 being high-falling, the one preceded by T3 being mid-rising or mid-level, and the one preceded by T4 being low-falling (Gao, 1980; Wang, 1996). However, the growth curve analyses showed that there were significant differences in tone shape for T1N, T3N, and T4N between the two groups. Compared to the neutral tones for the TD group, those for the ASD group were exhibiting overall flatter tonal contours.

### Tone error analysis

For the lexical tones, since the TD children performed almost perfectly, with only two T1 tokens being judged as errors, a generalized linear mixed-effects model was conducted only on the ASD children’s lexical tone errors using the lme4 package in R (Bates et al., 2015), with *p*-values calculated by the lmerTest package (Kuznetsova et al., 2017). The ASD children’s error data were treated as a binomial dependent variable, with correct responses coded as 1 and incorrect responses coded as 0. Tone (T1, T 2, T3, T4) was entered as a fixed factor, with T1 serving as the baseline. Participant and Item were included as random factors.

**Figure 6:**
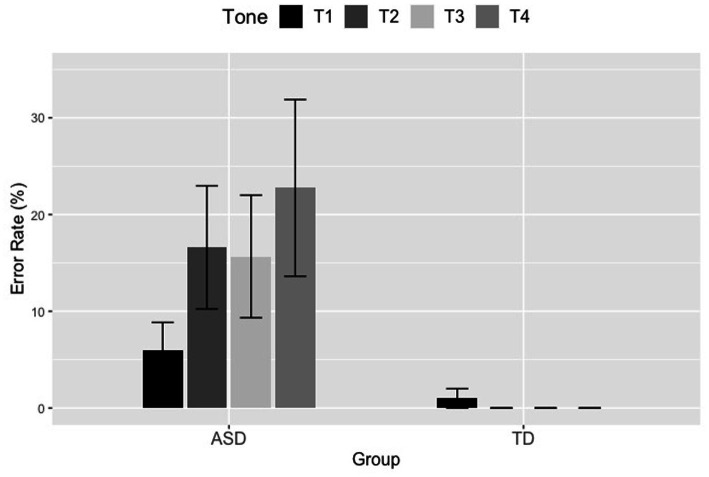
Tone error rates of the four lexical tones produced by the ASD and TD children in the first syllable position. The error bar indicates the 95% confidence interval.

Results of this model showed that the ASD children produced significantly more T2, T3, and T4 errors than T1 errors (T1 vs. T2: *β* = −2.065, *SE* = 0.471, *z* = −4.388, *p* < 0.001; T1 vs. T3: *β* = −2.109, *SE* = 0.473, *z* = −4.463, *p* < 0.001; T1 vs. T4: *β* = −2.044, *SE* = 0.473, *z* = −4.323, *p* < 0.001). To further compare the effects of different lexical tones, we re-ran this model but used T2 and T3 as the baseline for each run. Additional results of these two runs demonstrated that the ASD children produced similar numbers of errors between T2 and T3 (*β* = −0.044, *SE* = 0.435, *z* = −0.101, *p* = 0.920), and between T2 and T4 (*β* = 0.022, *SE* = 0.439, *z* = 0.049, *p* = 0.961), and between T3 and T4 (*β* = 0.065, *SE* = 0.437, *z* = 0.150, *p* = 0.881). The error results of lexical tones are shown in Figure 6.

For the neutral tones, a series of generalized linear mixed effects models were conducted on the ASD and TD children’s neutral tone errors using the lme4 package in R (Bates et al., 2015), with p-values calculated by the lmerTest package (Kuznetsova et al., 2017). Participants’ errors were deemed binomial data, with correct responses coded as 1 and incorrect responses coded as 0. Group (ASD, TD), Tone (T1N, T2N, T3N, T4N), and their interaction were included as fixed factors, with ASD and T1N serving as the baseline for Group and for Tone, respectively. Participant and Item were entered as random factors. Likelihood ratio tests using forward stepwise selection were employed to determine the best model, which was defined as the model containing the most fixed factors and fit significantly better than the one with one less variable. Results of the likelihood ratio tests showed that the model with Group, Tone, and their interaction could account for significantly more variance of the error data than the one without the interaction (*χ^2^*(3) = 37.890, *p* < 0.001), thus, determined as the optimal model (Errors ~ Group * Tone + (1|Participant) + (1|Item)).

Since the interaction effect between Group and Tone indicated that the error patterns of the four neutral tones differed significantly across the two groups of children, two generalized linear mixed-effects models were conducted, one within each participant group, to further investigate the error patterns of neutral tones for the ASD and TD children. For the two models, participants’ error data were set as a binomial dependent variable, with correct responses coded as 1 and incorrect responses coded as 0. Tone (T1N, T2N, T3N, T4N) was set as the fixed factor, with T1N treated as the baseline to which the other three neutral tones were compared. Participant and Item were entered as random factors.

Results of the ASD group showed that T2N elicited significant more errors than T1N (*β* = −1.183, *SE* = 0.316, *z* = −3.741, *p* < 0.001), and T3N yielded marginally significantly more errors than T1N (*β* = −0.630, *SE* = 0.324, *z* = −1.947, *p* = 0.052). However, T1N and T4N produced similar numbers of errors (*β* = −0.525, *SE* = 0.327, *z* = −1.609, *p* = 0.108). To further compare the effects of different neutral tones, we re-ran this model, but set T2N and T3N as the baseline for each run. Additional results of these two runs showed that T2N elicited significantly more errors than T4N (*β* = 0.657, *SE* = 0.297, *z* = 2.216, *p* = 0.027), and marginally significantly more errors than T3N (*β* = 0.553, *SE* = 0.292, *z* = 1.890, *p* = 0.059). Moreover, T3N and T4N yielded similar numbers of errors (*β* = 0.105, *SE* = 0.306, *z* = 0.342, *p* = 0.732).

**Figure 7:**
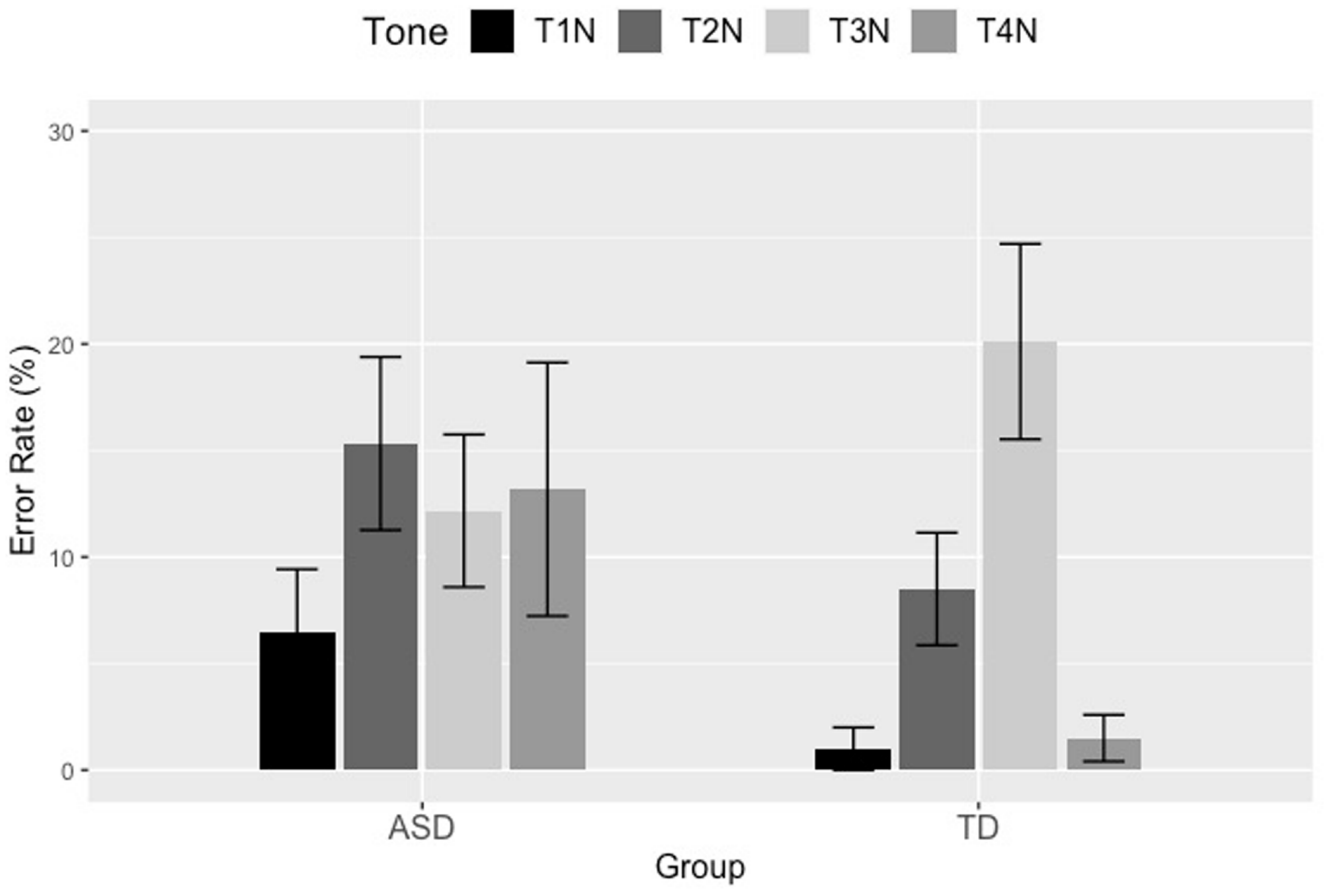
Tone error rates of the four neutral tones produced by the ASD and TD children in the second syllable position. The error bar indicates the 95% confidence interval.

Results of the TD group revealed that both T2N and T3N elicited significantly more errors than T1N (T1N vs. T2N: *β* = −2.338, *SE* = 0.772, *z* = −3.027, *p* = 0.002; T1N vs. T3N: *β* = −3.510, *SE* = 0.758, *z* = −4.628, *p* < 0.001), while T1N and T4N produced similar numbers of errors (*β* = −0.424, *SE* = 0.932, *z* = −0.455, *p* = 0.649). To further compare the errors of the other neutral tone pairs, we re-ran this model twice, with T2N and T3N entered as the baseline for each run. Results of the two runs revealed that T3N elicited significantly more errors than T2N (*β* = −1.172, *SE* = 0.352, *z* = −3.334, *p* < 0.001). T2N produced significantly more errors than T4N (*β* = 1.914, *SE* = 0.656, *z* = 2.920, *p* = 0.003), and T3N yielded significantly more errors than T4N (*β* = 3.086, *SE* = 0.639, *z* = 4.833, *p* < 0.001). The error results of neutral tones are plotted in Figure 7.

## Discussion

This study investigated the pitch patterns of the four lexical tones and the neutral tones following the preceding lexical tones produced by Mandarin-speaking children with ASD and their age-matched TD peers. The present results indicated that the ASD children have already developed the four lexical tones as well as neutral tone items, since the observed tonal contours of lexical tones and those of neutral tones are in line with previous research (Gao, 1980; Wang, 1996). However, in terms of the height and shape of lexical tones, we observed significant differences in tone height for T1 and T4, and in tone shape for T3 between the ASD group and the TD group, although T2 showed no difference between the two groups of children; likewise, for the tone patterns of neutral tones, we identified no difference in T2N, but found a significantly shallower tone shape of T1N, T3N, and T4N in the ASD group than in the TD group.

These differences observed by the comparison between the ASD children and their age-matched TD children pose a possibility that the atypical acoustic pattern in the ASD group could be partially due to the suppression of the F0 range. As shown in the present results, for the ASD children, the pitch pattern for lexical and neutral tones was realized by a narrower F0 range and a relatively shallower tonal shape. The shallower tonal contours for the Mandarin-speaking children with ASD in this study may be a result of the ASD children’s high sensitivity to acoustic details, so that they do not need to produce tones as acoustically distinct as the TD children do (Bonnel et al., 2003; Russo et al., 2008; Wang et al., 2017). Consistent with Yu and colleagues’ study (2015) that explored different neural processing data for lexical tones in the ASD group, the present study also found that ASD children tended to present inappropriate suppression of the overall F0 range. Wang et al. (2017) also revealed larger between-category mismatch negativity (MMN) than within-category MMNs for the TD group, while for the ASD group, comparable MMN effects were found for the two types of contrasts, indicating that categorical perception of lexical tones may be less evident for speakers with ASD. Additionally, Russo et al. (2008) raised the possibility that it might be the atypicality in the audio-vocal system that leads to a disturbance on the F0 feedback from their own speech, thus resulting in deficits in pitch regulation. Therefore, due to deficits in categorical perception and audio-vocal feedback, ASD individuals tend to create less distinct tones than TD individuals.

It is worth noting that among all four lexical tones, only T2 revealed no difference between the ASD group and the TD group. As displayed in Figure 2, T2 possessed the middle register in the pitch range, while T1 and T4 started from the high register, and T3 ended in the low register. The aberrant height and shape of T1, T3, and T4 showed that they were squeezed from the edges of the F0 range. These data, thus, showed the pattern that for the ASD individuals, the peripheral tones, namely, the high and low tones, were most strongly impacted, but the middle tone, in this case T2, was immune from being impacted. Similarly, the production of T2N in the ASD group was also patterned with that in the TD group. A possible reason for this phenomenon might be that T2N could be the continuation of T2. If the contour of T2 was not significantly different between the two groups, it was likely that the contour of T2N was not significantly different between the two groups either. Future studies should be conducted to further investigate the productions of T2 and T2N between ASD and TD children, and test the hypotheses of F0 range suppression and F0 continuation.

Nevertheless, one thing that both the previous and present results point to, regardless of whether the language is tonal or non-tonal, is that there are significant acoustic differences in pitch patterns between the ASD and TD groups. More specifically, the ASD group was reported to sound monotonous or machine-like (Pronovost et al., 1966; Goldfarb et al., 1972; Fay and Schuler, 1980; Paccia and Curcio, 1982; Baltaxe et al., 1984; Baltaxe and Guthrie, 1987; Kaland et al., 2013), and produced smaller F0 variation of intonation compared to the neurotypical controls (Grossman et al., 2010; Kaland et al., 2012, 2013; Nakai et al., 2014). Therefore, the conclusion can be made safely that the atypicality of F0 realization in pitch patterns is one of the typical symptoms of ASD across languages.

Additionally, for the error rates of lexical tones, the TD children performed almost perfectly (with only two T1 tokens being judged as incorrect T1), implying that those children have indeed achieved mastery in Mandarin tone as reported in previous studies (Chao, 1951; Li and Thompson, 1977; Clumeck, 1980; Zhu and Dodd, 2000; Zhu, 2002; Si, 2006). Given that the children in this study were around 6.5 years of age at the time of testing, they were expected to produce the four lexical tones correctly. However, we found that the ASD children yielded higher error rates of lexical tones than the TD children, as shown in Figure 6. Moreover, ASD children produced significantly more T2, T3, and T4 errors than T1 errors. Since Mandarin-speaking children with ASD were shown to have pitch processing deficits around 9.3 years of age in Yu et al. (2015) and lack the categorical perception of lexical tones around 10.4 years of age in Wang et al. (2017), it is highly possible that the ASD children in the present study have tone perception difficulties that impact their tone acquisition. For the error rates of neutral tones, we found that the ASD children presented a similar error pattern as the TD groups, with both groups producing similar numbers of errors between T1N and T4N that elicited fewer errors than T2N and T3N. Such findings provide further evidence in support of the universal acquisition principle of pitch (e.g., Halliday, 1975; Menn, 1976; Crystal, 1979), that is, level and falling tones (i.e., T1 and T4) are thought to be the easiest tones to acquire since level tones and falling tones are easier to pronounce than rising and dipping tone (i.e., T2 and T3). As mentioned in the introduction, T2 and T3 are more difficult to acquire due to their rising and falling-rising tonal contours. Moreover, T2 and T3 contours are similar and easy to be confused with each other (Li and Thompson, 1977) due to the phonetic similarity between the two tones. In addition, T3 can alternate with T2 *via* the third tone sandhi rule, in which case, T3 turns into T2 when followed by another T3, which further leads to the confusion between T2 and T3 (Chao, 1951; Li and Thompson, 1977; Clumeck, 1980; Chen, 2000; Zhu and Dodd, 2000). Given the acquisitional, phonetic, and phonological reasons, T2 and T3 should elicit more errors compared to the other two tones, as presented in this study. Besides, we found that a large proportion of the incorrect tokens in T3N were merely mispronunciations in this study, which was to a great extent due to the complex realization of T3 itself. T3 as a lexical tone has several surface variants (Li and Thompson, 1977). T3 can be realized as a low falling tone when followed by T1, T2, and T4 (half-third sandhi), as a high-rising tone when followed by another T3, as a falling-rising tone when being in the utterance-final position and in isolation (e.g., Lin, 1996; Shih, 1997; Peng, 2000; Duanmu, 2007; Lin, 2007). Even as a neutral tone, T3N can be realized as both mid-level and mid-rising. Additionally, the rising shape of T3N may take greater physiological efforts than the falling shape of T1N, T2N, and T4N (Ohala and Ewan, 1973). These reasons may partially explain why a large proportion of incorrect T3N tokens were mispronunciations.

Based on the abovementioned findings, we thus propose that such pitch processing impairments should impact different tones in different ways since it may interact with some other factors such as universal acquisition principles of pitch, similarities among different tones, and tone sandhi. Apart from the deficits in pitch processing that could be responsible for the tone patterns of ASD children, the atypical tonal contours produced by the ASD group might also be due to imitation deficit, which is unique to ASD and could be related to social and communication deficits (Rogers et al., 2003). Green and Tobin (2009) also pointed out that for the ASD group, pitch variation was not always optimized for communicative intent. For example, those with ASD appeared to use a restricted number of prosodic contours in their utterances. This study utilized a pronunciation task in which the children were asked to read the target stimuli they heard out loud, which involved imitation and social communication and may negatively influence the results. Therefore, more research should also be conducted to investigate the relationship between social communication skills and tone production for ASD speakers in the future.

## Conclusion

The present findings of the acoustic analyses suggest that Mandarin-speaking children with ASD, in general, produced Mandarin tones well, with each lexical tone and neutral tone having its own distinct tonal contour. However, the tones produced by the ASD children were shallower than those produced by the TD children. These data, together with the previous findings on non-tonal languages, indicated that the atypical F0 realization for speakers with ASD may be language-independent. Our error data further show that although the two groups of children yielded similar error rates for the neutral tones, the ASD children produced significantly higher error rates for the lexical tones. The pattern of tone errors can be partially explained by universal acquisition principles of pitch, similarities among different tones, and tone sandhi. We further concluded that apart from the deficits in pitch processing that could be responsible for the atypical tone patterns of the ASD children, the atypical tonal contours produced by the ASD group might also be due to imitation deficits. Still, the interaction of social communication skills and tone productions, whether the underlying tone of the second morpheme influences the performance of the neutral tone, and the nature of the tone errors, are left to be examined in future research.

## Data availability statement

The raw data supporting the conclusions of this article will be made available by the authors, without undue reservation.

## Ethics statement

The studies involving human participants were reviewed and approved by the Ethics Committee of School of Psychological and Cognitive Sciences at Cangzhou Normal University. The patients/participants provided their written informed consent to participate in this study.

## Author contributions

KX: conceptualization, writing–original draft, writing–review editing, and supervision. JY: investigation, funding acquisition, and data collection. CM: data curation, visualization, and writing–review and editing. XC: data curation and writing–review and editing. Y-FC: conceptualization, formal analysis, and writing–original draft. All authors contributed to the article and approved the submitted version.

## Funding

This research was partially supported by China Postdoctoral Science Foundation under the Project of Characteristics of the Speech Prosody Produced by Chinese Autistic Children (2022M711117), Hebei Social Science Fund under the project of Experimental Research in Rhythmic Characteristics on Discourse of Autistic Children (HB20YY006), and a grant from 2022 Individualized Programs for Original Creative Scientific Research of Fudan University (IDH4307370-13).

## Conflict of interest

The authors declare that the research was conducted in the absence of any commercial or financial relationships that could be construed as a potential conflict of interest.

## Publisher’s note

All claims expressed in this article are solely those of the authors and do not necessarily represent those of their affiliated organizations, or those of the publisher, the editors and the reviewers. Any product that may be evaluated in this article, or claim that may be made by its manufacturer, is not guaranteed or endorsed by the publisher.
